# Supplementary material for: The interactome of intact mitochondria by cross-linking mass spectrometry provides evidence for coexisting respiratory supercomplexes
Source: Mol Cell Proteomics. 2017 Dec 8;17(2):216–32. doi: 10.1074/mcp.RA117.000470 (PMC5795388; doi:10.1074/mcp.RA117.000470)
Supplement: Supplemental Data [file supp_RA117.000470_133922_0_supp_23978_fzffwf.zip › spectra_annotation/mito_spectra_annotation/122-1-4-1-5-1.pdf]

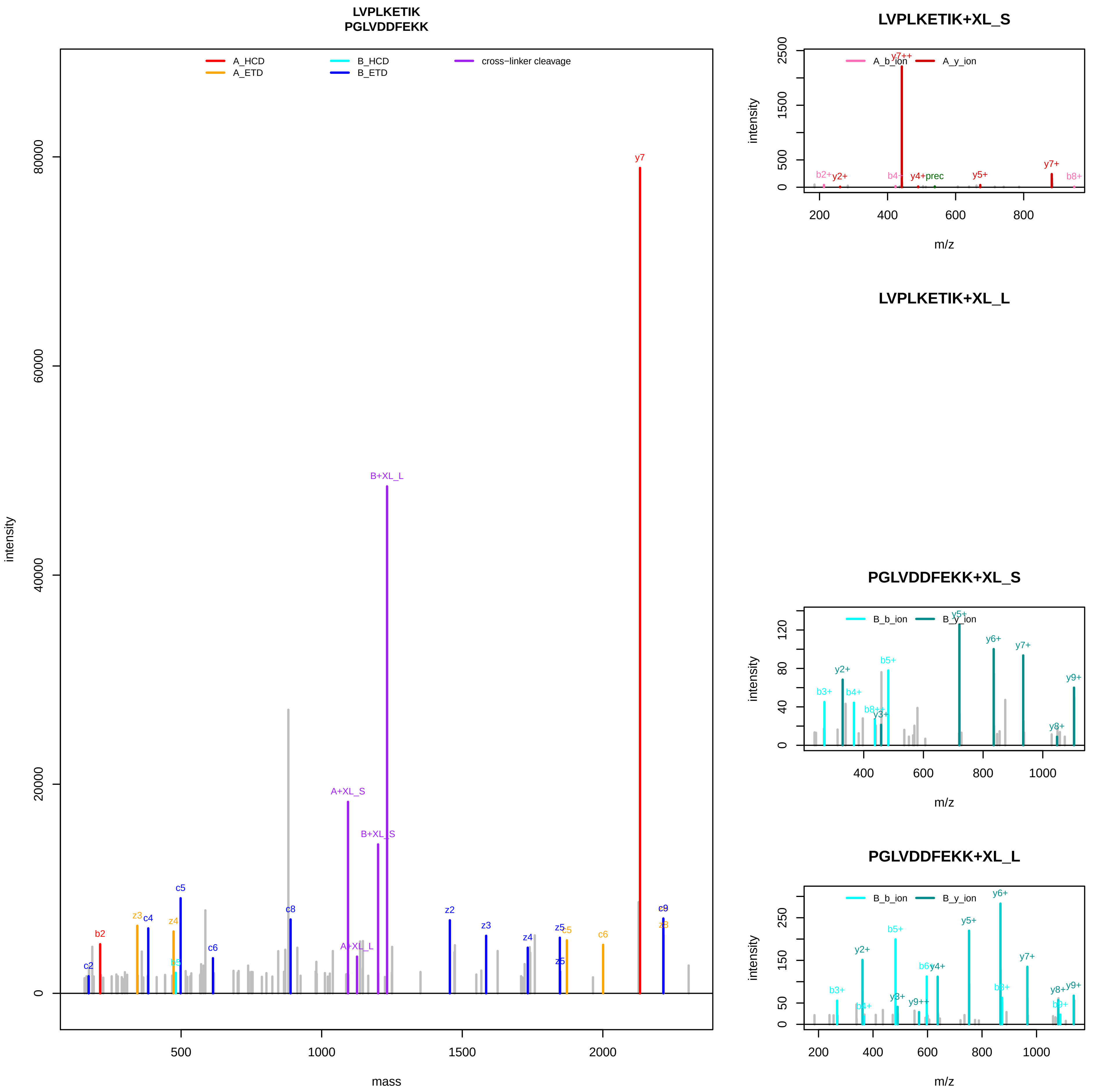
LVPLKETIK
PGLVDDFEKK
A_HCD
A_ETD
B_HCD
B_ETD
cross−linker cleavage
y7
B+XL_L
A+XL_S
B+XL_S
A+XL_L
c2
b2
z3
c4
z4
c5
c6
c8
z2
z3
z4
z5
c5
c6
c9
z8
intensity
mass
LVPLKETIK+XL_S
A_b_ion
A_y_ion
y7++
b2+
y2+
b4+
y4+
prec
y5+
y7+
b8+
m/z
LVPLKETIK+XL_L
PGLVDDFEKK+XL_S
B_b_ion
B_y_ion
y5+
y6+
y7+
y2+
b5+
b3+
b4+
y3+
y9+
y8+
PGLVDDFEKK+XL_L
y6+
y5+
b5+
y2+
b6+
y4+
y7+
b3+
b4+
y3+
y9++
b8+
y8+
y9+
b9+
